# Impacts of hoof disorders on milk yield in cattle: a systematic review with meta-analysis

**DOI:** 10.1007/s11250-026-04967-1

**Published:** 2026-03-05

**Authors:** José Leôncio Delmondes Pereira Freitas, Paulo Henrique Conceição Costa, Ana Luiza e Silva Santos Soares, Luana Moura Delmondes Freitas, Luís Fernando Batista Pinto

**Affiliations:** Animal Sciences Department, Federal University of Bahia, Salvador, Bahia Brazil

**Keywords:** Cows, Lesion, Lameness, Hoof, Milk

## Abstract

Hoof disorders (HD) may affect milk yield (MY) in dairy cows, but previous studies found a considerable variation of this HD effect. This study aimed to conduct a comprehensive systematic review to estimate pooled effect sizes of HD on MY in dairy cows based on meta-analysis. Initially, searches in PubMed, Scopus, and Web of Science databases allowed the identification of 4,074 papers. After excluding duplicates and screening titles and abstracts, 17 papers reporting multiple estimates of mean MY differences between HD-affected and healthy cows were identified. Standardized mean differences (SMD) were calculated, as papers used different MY units. All subgroup meta-analyses were carried out using the study as a random effect. In primiparous cows, the pooled SMD in sub-clinical and clinical condition were − 0.0304 (95%CI: -0.0607 to -0.0001) and − 0.0580 (95%CI: -0.1063 to -0.0097), respectively. Multiparous cows with sub-clinical and clinical HD conditions had pooled SMD of -0.1015 (95%CI: -0.1670 to -0.0360) and − 0.1556 (95%CI: -0.2119 to -0.0993), respectively. The pooled SMD for digital dermatitis (DD) -0.0673 (95%CI: -0.0886 to -0.0460), sole ulcer (SU) -0.0795 (95%CI: -0.1210 to -0.0380), and white line disease (WLD) -0.0870 (95%CI: -0.1524 to -0.0217) affected cows were also estimated. The heterogeneity between studies varied from low to moderate but was insignificant (p-value > 0.05) based on the Q-test. HD negatively impacts MY in primiparous or multiparous cows in clinical and sub-clinical conditions. Based on the current literature, it can not be stated that SU-, DD-, and WLD-affected cows show different daily MY losses.

## Introduction

Hoof disorders (HD) have incidence rates varying from small to high values in dairy cattle. Some studies reported incidence rates < 10% (Kocak and Ekiz 2006; Miciński et al. 2009; Rajala-Schultz et al. 1999), while many studies estimated values between 10% and 40% (Bicalho et al. 2008; Charfeddine and Pérez-Cabal 2017; Olechnowicz and Jaśkowski 2010; van den Borne et al. 2022). Even incidences of 70% have also been reported (Green et al. 2002). Therefore, HD is a source of concern for milk producers, as it causes economic losses and negatively impacts cows’ welfare (Bruijnis et al. 2012a, b).

Hoof clinical evaluation (Amory et al. 2008; Green et al. 2010; Kocak and Ekiz 2006; Miciński et al. 2009; Pavlenko et al. 2011; Rajala-Schultz et al. 1999; Singh et al. 2011) or visual scoring of the cows while standing and walking (Thomsen et al. 2008) are methods that allow identification HD-affected cows. Depending on the injury level, HD can be classified as mild, moderate, or severe (Charfeddine and Pérez-Cabal 2017; Warnick et al. 2001). Cows with moderate or severe HD usually show lameness, characterized by locomotion difficulties. Lame cows may cause substantial economic losses to dairy systems due to factors such as cow veterinary treatment, trimmer, extra labor, premature culling, additional days open, and milk yield (MY) reductions. Charfeddine and Pérez-Cabal (2017) estimated annual costs ranging from €53 per dermatitis-affected cow with mild lesions to €622.3 per sole ulcer (SU) affected cows with severe lesions.

Several studies estimated the HD effect on MY, comparing HD-affected with healthy cows. SU and hoof rot (HR) affected cows had MY losses ranging from 0.9 to 2.5 kg/day and from 0.4 to 1.2 kg/day, respectively (Warnick et al. 2001). White line disease (WLD) also caused MY losses, with values ranging from 0.64 to 2.18 kg/day (Amory et al. 2008). Cows with double sole (DS) had a mean MY loss of 1.76 kg/day (Green et al. 2010). Dairy cows with digital dermatitis (DD) showed a considerable (5.5 kg/day) mean MY loss (Pavlenko et al. 2011). MY losses can occur even before HD diagnosis, i.e., in a sub-clinical condition (Green et al. 2002; Rajala-Schultz et al. 1999), and many cows continue to experience MY losses for many days after treatment (Green et al. 2010; Pavlenko et al. 2011).

Although MY losses have been reported in many studies that analyzed HD-affected cows, there was no consensus regarding the effect size. In some cases, the MY losses were even insignificant (Kocak and Ekiz 2006). The MY loss differences across studies may result from HD severity, experimental design, or sampling error. Therefore, a meta-analysis of the many previous results may produce pooled and robust estimates of MY loss that better describe the impacts of HD on MY in dairy cows. Thus, the primary objective of this study was to conduct a comprehensive systematic review to estimate pooled effect sizes of HD on MY in dairy cows based on meta-analysis.

## Materials and methods

### Systematic review

All data analyzed in the present systematic review are available in previously published scientific papers. No ethics committee analysis was necessary as neither animal nor human experiments were carried out. The key question guiding this systematic review was: What is the impact of HD on MY in dairy cows? The Pubmed, Scopus and Web of Science databases were used for the systematic review. The search strategies were as follows: (1) PUBMED: (Cattle[Title/Abstract] OR Bovine[Title/Abstract] OR Cow[Title/Abstract] OR Heifer[Title/Abstract] OR Calf[Title/Abstract] OR Dairy[Title/Abstract]) AND (milk[Title/Abstract] OR production[Title/Abstract] OR reproduction[Title/Abstract] OR fat[Title/Abstract] OR protein[Title/Abstract] OR culling[Title/Abstract]) AND (lameness [Title/Abstract] OR digital dermatitis[Title/Abstract] OR heel horn erosion[Title/Abstract] OR interdigital dermatitis[Title/Abstract] OR interdigital hyperplasia[Title/Abstract] OR sole hemorrhage[Title/Abstract] OR sole ulcer[Title/Abstract] OR toe ulcer[Title/Abstract] OR white line disease[Title/Abstract]); (2) SCOPUS: TITLE-ABS-KEY (cattle OR bovine OR cow OR heifer[ OR calf OR dairy) AND TITLE-ABS-KEY (milk OR production OR reproduction OR fat OR protein OR culling) AND TITLE-ABS-KEY (lameness) OR TITLE-ABS-KEY (digital AND dermatitis) OR TITLE-ABS-KEY (heel AND horn AND erosion) OR TITLE-ABS-KEY (interdigital AND dermatitis) OR TITLE-ABS-KEY (interdigital AND hyperplasia) OR TITLE-ABS-KEY (sole AND hemorrhage) OR TITLE-ABS-KEY (sole AND ulcer) OR TITLE-ABS-KEY (toe AND ulcer) OR TITLE-ABS-KEY (white AND line AND disease); and (3) WEB OF SCIENCE: cattle OR bovine OR cow OR heifer OR calf OR dairy (Topic) and milk OR production OR reproduction OR fat OR protein OR culling (Topic) and lameness OR digital dermatitis OR heel horn erosion OR interdigital dermatitis OR interdigital hyperplasia OR sole hemorrhage OR sole ulcer OR toe ulcer OR white line disease (Topic). All these databases were accessed on July 18, 2023.

**Fig. 1:**
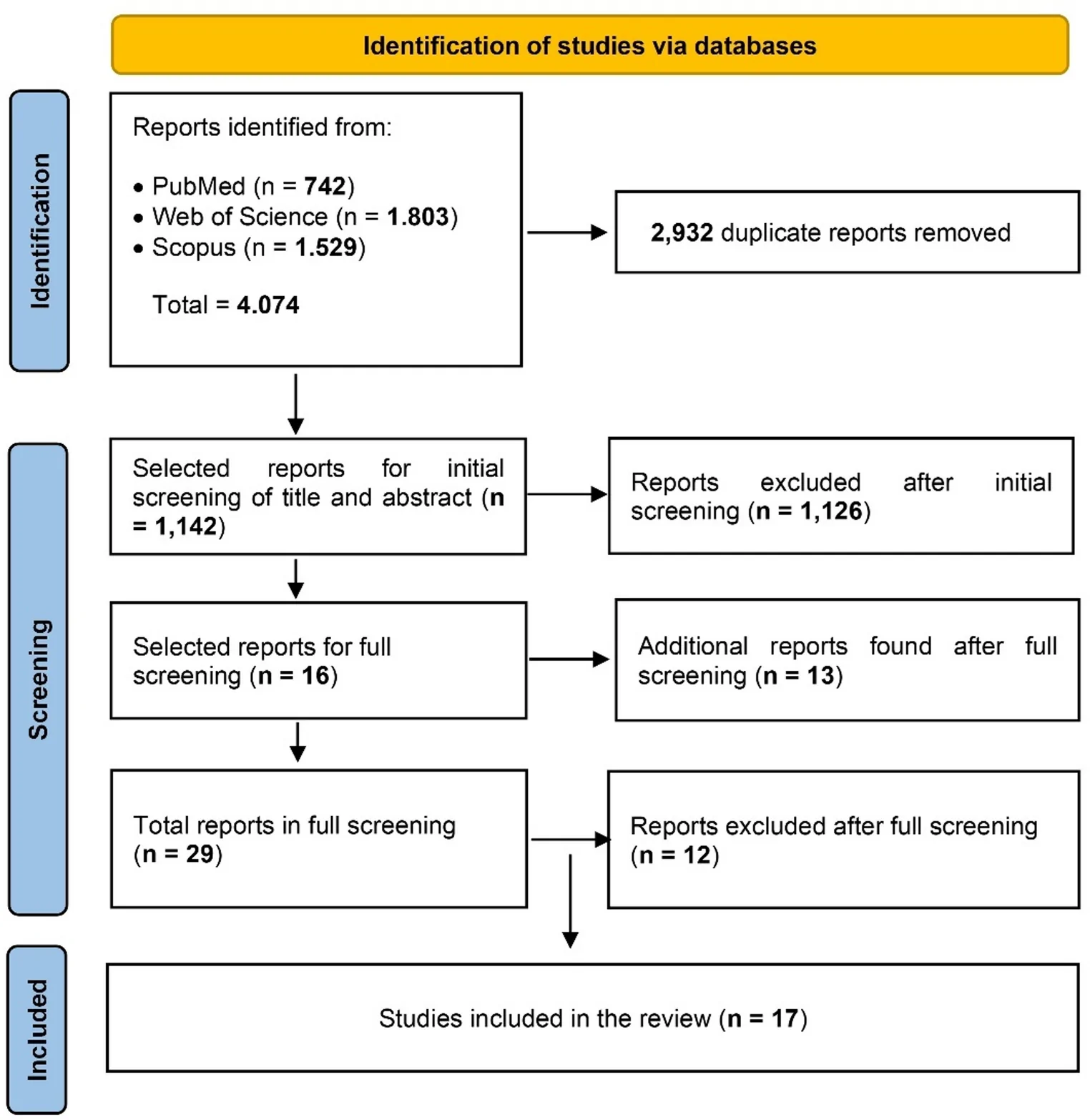
PRISMA diagram of the record flow, adapted from (Page et al., 2021)

The search strategies found 4,074 documents (Fig. 1). They were exported for subsequent analysis into the Rayyan tool (Ouzzani et al. 2016), where 2,932 duplicate documents were detected. After excluding duplicates, 1,142 documents were eligible for initial screening, which consisted of reading the title and abstract of each paper. Then, all papers that did not respond to the key question and papers written in other than English, Portuguese, or Spanish languages were excluded. After this initial screening, 16 papers were eligible for the full screening. These papers revealed 13 additional documents that were not found by our search strategies, which were also included for full screening. After a full screening of 29 papers, 17 of them were selected. Then, data from selected papers were extracted into a spreadsheet, which included: bibliographic reference, year of publication, country, breed, lactation order, time of diagnosis, type of HD (sole ulcer, digital dermatitis, white line disease, etc.), HD severity, total sample size, number of HD-affected and healthy cows, effect size (i.e., mean MY of HD-affected and healthy cows or mean MY differences between these groups), and standard error (SE) of the effect size (or statistics that allowed the SE calculation).

### Descriptive statistics

All effect sizes previously reported in units other than kg/day were converted to kg/day to perform a descriptive analysis of previously reported effect sizes, including identifying minimum and maximum values​, estimating mean, median, and standard deviation, and plotting distribution. All effect sizes ​​outside the upper and lower boxplot bounds, i.e. outside the whisker, were assumed to be outliers and removed.

### Meta-analysis

First, standardized mean differences (SMD) between HD-affected and healthy cows were calculated for each study, as different MY units were used across studies. The SMD and its SE were calculated as suggested by Harrer et al. (2022):

$$SMD=\frac{{\stackrel{-}{x}}_{a}-{\stackrel{-}{x}}_{b}}{\sqrt{\frac{{S}_{a}^{2}\left({n}_{a}-1\right)+{S}_{b}^{2}\left({n}_{b}-1\right)}{{n}_{a}+{n}_{b}-2}}}$$

where ${\stackrel{-}{x}}_{a}$ and ${\stackrel{-}{x}}_{b}$ are the mean MY of HD-affected and healthy cows, respectively; ${S}_{a}^{2}$ and ${S}_{b}^{2}$ are the variances of HD-affected and healthy cows, respectively; ${n}_{a}$ and ${n}_{b}$ are the sample size of HD-affected and healthy cows, respectively. Moreover, the SE of each SMD was calculated as:

$${SE}_{SMD}=\sqrt{\frac{{n}_{a}+{n}_{b}}{{n}_{a}{n}_{b}}+\frac{{SMD}^{2}}{2({n}_{a}+{n}_{b})}}$$

The meta-analyses were performed using the R “meta” package (Balduzzi et al. 2019), based on the model:

$${\widehat {\rm{\theta }}_k} = \,\widehat {\rm{\theta }}\, + \,\beta {X_k}\, + \,{\varsigma _k}\, + \,{\varepsilon _k}$$

where ${\widehat{\theta}}_{k}$ is the SMD estimate in the study k, $\widehat{\theta}$ is the pooled SMD estimated by meta-analysis, *β* is the regression coefficient for the fixed effect *X*, ${\zeta}_{k}$ is the random effect of study k, for which was assumed $\varsigma k\,\sim N(0,{\tau ^2})$, where ${\tau}^{2}$ is the variance caused by the between study heterogeneity (BSH); and ${\epsilon}_{k}$ is random residual effect, for which was assumed ${\epsilon}_{k}\, \sim N(0,{\sigma}^{2})$, where ${\sigma}^{2}$ is the residual variance. The pooled SMD estimate was calculated as:

$$\widehat{\theta}=\frac{\left({\sum}_{k=1}^{n}{\widehat{\theta}}_{k}{w}_{k}^{*}\right)}{{\sum}_{k=1}^{n}{w}_{k}^{*}}$$

where ${w}_{k}^{*}=\frac{1}{\left(\frac{{\sigma}_{e}^{2}}{n}+{\tau}^{2}\right)}$ is the weight of study *k*. The Restricted Maximum Likelihood (REML) method estimated the random effects (${\tau}^{2}$, ${\sigma}^{2}$) in the model, which is a suitable method when continuous effect sizes are analyzed (Harrer et al. 2022). The Hartung-Knapp method(Hartung and Knapp 2001) calculated the SE of the pooled SMD estimate, as it generally produces slightly larger confidence intervals than other methods and is especially recommended when few studies are included in the meta-analysis or when there is a significant BSH (Harrer et al. 2022).

The previous model was used for subgroup meta-analyses (Harrer et al. 2022), which fitted a single categorical fixed effect in X. Three subgroup meta-analyses were performed as follows: (1) pooled SMDs were estimated for two HD conditions (clinical or sub-clinical) in primiparous cows, (2) like the previous one but using data from multiparous cows, and (3) pooled SMDs were estimated based on three HD reasons (SU, DD, and WLD).

### Between studies heterogeneity

The Q test(Cochran 1954) was used to distinguish the sampling error from the BSH error. This test can be calculated as:

$$Q={\sum}_{k=1}^{n}{w}_{k}^{*}{\left({\widehat{\theta}}_{k}-\widehat{\theta}\right)}^{2}$$

The *Q* has a chi-square distribution with *(k-1)* degrees of freedom, where *k* is the number of studies in the meta-analysis. If *Q* is statistically equivalent to *(k-1)*, i.e., Q-test p-value > 0.05, then the differences between studies are a consequence of sampling error, and BSH is negligible. On the other hand, when *Q* is statistically different to *(k-1)*, there is a significant error caused by BSH. A second *Q* test was performed in subgroup meta-analysis to evaluate the null hypothesis that pooled SMD estimates do not differ across fixed effect classes (Harrer et al. 2022). A 5% significance level was used in all hypothesis tests.

Another metric used to evaluate BSH was the heterogeneity index (${I}^{2}$), which describes the percentage of the total variation across studies that is due to BSH rather than sampling error. The ${I}^{2}$ was calculated using the Cochran’s *Q* value as follows: ${I}^{2}=\left(\frac{Q-(K-1)}{Q}\right)\times100$, where ${I}^{2}$ values around 25%, 50%, and 75% can be classified as low, moderate, and high BSH, respectively (Higgins and Thompson 2002).

The BSH can be caused by one or more studies with extreme effect sizes (outliers), which can affect the pooled SMD estimate. Therefore, the {find.outliers} function of the R “dmetar” package (Harrer et al. 2022) was used to check outlier SMD estimates. The {find.outliers} function suggests as an outlier any SMD estimate with a 95% confidence interval (95% CI) that does not overlap (at least partially) the 95% CI of the pooled SMD estimate.

In addition, it is also essential to know whether the pooled SMD estimate is robust, i.e., it does not depend heavily on a single very influential study. The {Influence} and (leave1out} functions, both from “metafor” R package (Viechtbauer 2010), were used to check influential SMD estimates. The {Influence} and (leave1out} functions performed multiple meta-analyses by omitting one SMD per time to identify influential SMD estimates (Viechtbauer 2010).

Subsequently, the Shapiro-Wilk test was used to verify whether the set of SMD estimates used in the meta-analyses had a normal distribution, which is an essential assumption in meta-analysis (Harrer et al. 2022). If Shapiro-Wilk p-value ​​was greater than 0.05, the null hypothesis is accepted and SMD estimates are assumed to be normally distributed.

Publication bias was assessed based on graphical analysis of funnel plots (Harrer et al. 2022), which was plotted using the {funnel} function from the “metafor” R package (Viechtbauer 2010). The X-axis of funnel plots shows the SMD estimates included in the meta-analysis, while the Y-axis shows the respective SE. Funnel plots are inverted, i.e., higher values ​​on the Y-axis indicate smaller SE. The funnel plot is an effective method that can be applied to any meta-analysis and is especially useful for meta-analyses with few estimates (Harrer et al. 2022). An asymmetry in the funnel plot suggests publication bias.

The main results of the meta-analyses, including pooled SMD estimate, its 95% CI, and the metrics used to assess heterogeneity (*Q* and ${I}^{2}$) were included in the forest plots, by using the function {forest} from R “metafor” package (Viechtbauer 2010).

## Results and discussion

### Countries and breeds

The studies analyzed in this review were carried out in many countries (Table 1), which are located mainly in temperate climate. Moreover, most studies used Holstein cows. Pavlenko et al. (2011) analyzed data from Holstein and Swedish Red cows together, i.e., they did not report results for each group separately. Rajala-Schultz et al. (1999) and Singh et al. (2011) evaluated the Ayrshire and Karan Fries dairy cows, respectively, while two studies did not specify the dairy breeds (Amory et al. 2008; King et al. 2017). Therefore, the HD effects on MY in many dairy cattle breeds, such as Jersey, Guernsey, and Girolando, remain unknown. In addition, HD effects in cows raised in tropical regions also remain poorly known. Evaluating other dairy breeds in different regions may help identify those more resilient/resistant, characterizing the HD effects on MY more consistently.

### Experimental groups

All the previous studies analyzed at least two experimental groups, i.e., HD-affected and healthy cows. The HD-affected group consisted of cows with no other diseases, such as mastitis or ketosis, to avoid confounding disease effects. Some studies separated HD-affected and healthy cows based on clinical examination of the hooves (Amory et al. 2008; Green et al. 2010; Kocak and Ekiz 2006; Miciński et al. 2009; Pavlenko et al. 2011; Rajala-Schultz et al. 1999; Singh et al. 2011). There were also studies that used visual analysis to separate HD-affected and healthy cows, where HD-affected cows showed arched back when standing and walking and an abnormal gait (Bicalho et al. 2008; Green et al. 2002; Hernandez et al. 2002).

Some studies assessed HD severity. Warnick et al. (2001) analyzed four experimental groups (healthy, and mild, moderate or severe HD-affected) by clinically examining the hooves of each cow but did not clearly describe how they separated the HD severity classes. Charfeddine and Pérez-Cabal (2017) formed three experimental groups (healthy, and mild or severe HD-affected), having diagnosed all superficial injuries that did not affect the deeper tissues as mild HD-affected, while severe HD-affected invaded the deeper tissues of the horn, resulting in sepsis.

Moreover, the HD severity was assessed based on locomotion scores, also called lameness scores (Hernandez et al. 2005; King et al. 2017; Olechnowicz and Jaśkowski 2010; Onyiro et al. 2008; van den Borne et al. 2022). A scoring system example can be found in Thomsen et al. (2008) as follows: 1 – Normal (the cow walks normally, and the back is flat, both when the cow is standing and when walking), 2 – Mild lame (the cow walks almost normally and the back is flat when it is standing, but arched when walking), 3 – Moderate lame (the cow has abnormal gait with short strides on at least one leg. The back is arched both when the cow is standing and walking, but an observer will not be able to identify which leg is affected), 4 – lame (cow is obviously lame on one or more legs and an observer will be able to identify which legs are affected), and 5 – severe lame (the cow is obviously lame on one or more legs and it is unable, unwilling, or very reluctant to bear weight on the affected leg). In the previous studies, the scores were usually assigned by a single person, who observed each cow while they were standing or walking on a flat, unobstructed area.

**Table 1:** Summary description of the 17 papers analyzed, including country, breed, sample size in HD-affected and healthy cows, unit of milk yield, statistical approach, and lactation order

References	Country	Breed	Sample size^1^	Unit	Statistical approach^2^	Lactation order^3^
Amory et al. (2008)	UK	NA	1188 (169 to 230)	kg/d	R	*
Bicalho et al. (2008)	USA	Holstein	603 (603)	kg/d	R	*
Borne et al. (2022)	Netherlands	Holstein-Friesian	995 (176 to 437)	kg/d	S	*
Charfeddine e Pérez-Cabal (2017)	Spain	Holstein	21,011 to 40,350 (121 to 9086)	kg/d	R	P/M
Green et al. (2002)	UK	Holstein-Friesian	270 (630)	kg/d	R	*
Green et al. (2010)	Chile	Holstein-Friesian	1427 to 1503 (132 to 208)	kg/d	R	*
Hernández et al. (2002)	USA	Holstein	364 (15 to 100)	lb/305d	S	*
Hernandez et al. (2005)	USA	Holstein	11 to 84 (74 to 212)	lb/305d	S	*
King et al. (2017)	Canada	NA	865 (353)	kg/d	S	*
Kocak and Ekiz (2006)	Turkey	Holstein	82 to 867 (10 to 86)	kg/305d	S	P/M
Miciński et al. (2009)	Poland	Holstein-Friesian	40 to 61 (7 to 47)	kg/305d	S	P/M
Olechnowicz and Jaśkowski (2010)	Poland	Holstein-Friesian	76 (56 to 92)	kg/120d	S	*
Onyiro et al. (2008)	Scotland	Holstein-Friesian	63 to 176 (4 to 117)	kg/d	S	P/M
Pavlenko et al. (2011)	Sweden	Swedish Red e Holstein	20 (10)	kg/d	R	*
Rajala-Schulyz et al. (1999)	Finland	Ayrshire	2255 to 5694 (61 to 185)	kg/d	R	P/M
Singh et al. (2011)	India	Karan Fries	67 (96)	kg/305d	S	*
Warnick et al. (2001)	USA	Holstein	211 to 871 (17 to 925)	kg/dia	R	P/M

^1^Number of healthy cows and (HD-affected cows); ^2^R and S indicate studies that used repeated measures over time and single milk yield records, respectively. ^3^An asterisk (*) indicates studies that fitted the calving order into the model but did not report the HD effect for each calving level; while P/M studies estimated HD effect for Primiparos/Multiparous separately

There was a variation across studies that used locomotion scores to define the experimental groups. Hernandez et al. (2005) grouped the cows into three scoring classes: cows showing scores ≤ 2 as non-lame, 3 as moderately lame, and ≥ 4 as lame. Van den Borne et al. (2022) considered scores < 2.25 as healthy cows, between 2.25 and 3.25 as moderately lame, while severe lameness cows had scores above 3.25. Onyiro et al. (2008) and King et al. (2017) formed only two groups, healthy (scores < 3) and HD-affected (scores ≥ 3) cows. This design variation across studies, as well as the possible differences in clinical or visual diagnostic, may cause BSH, which was accounted by fitting study as a random effect in the model.

### Sample size

The previous studies analyzed small, medium, and large samples (Table 1). The smallest study analyzed groups with 20 healthy cows and 10 HD-affected cows (Pavlenko et al. 2011). On the other hand, the largest study had between 21,011 and 40,350 healthy cows and from 121 to 9,086 HD-affected cows (Charfeddine and Pérez-Cabal 2017). Sample size plays a very important role in this type of study, since the significance of the hypothesis tests depends on the test power, which in turn is determined by the sample size. Thus, it is expected that very small studies will be able to identify only very large effect size as significant, while large studies may find small effect size as significant. Differences in sampling are weighted by meta-analysis, as the studies with lower SE receive higher weights than studies with higher SE.

### Milk yield units

Previous studies recorded MY in different ways (Table 1). Many studies reported daily MY (in kg/day), while some studies reported MY for an entire lactation (in kg/305 days) (Miciński et al. 2009). There was also a study that evaluated the impact of lameness on total milk yield during the first 120 days of lactation (Olechnowicz and Jaśkowski 2010), as the incidence of HD-affected cows was higher in this period. Studies that evaluated daily milk yield in lb/305 day were also found (Hernandez et al. 2002, 2005). These differences across studies prevent using mean differences between groups (HD-affected and healthy cows) as the outcome to be meta-analyzed. In this case, it is necessary to obtain the SMD, which consists of dividing the mean difference (MD) by the pooled standard deviation (SD). SMD should be interpreted as the MY difference between HD-affected and healthy cows in SD equivalents. For example, a SMD = -1 indicates that the MY from HD-affected cows was one (1.0) SD lower than the MY of healthy cows.

### Statistical approach

The previous studies used two main statistical approaches. Longitudinal data analysis was mainly employed to evaluate multiple test-day records (Amory et al. 2008; Bicalho et al. 2008; Charfeddine and Pérez-Cabal 2017; Green et al. 2002, 2010; Pavlenko et al. 2011; Rajala-Schultz et al. 1999; Warnick et al. 2001). This approach allows modeling the complex structure of correlations between MY records in the same cow at different times across lactation. Furthermore, test-day records can reflect cow performance closest to the HD diagnostic day, which is probably better than total milk yield from an entire lactation. Other studies used a single MY record per cow, i.e., not repeated measures. In this case, some studies used daily MY recorded at some point across the lactation, usually close to the HD diagnostic day (King et al. 2017; Onyiro et al. 2008; Van den Borne et al. 2022), total MY adjusted for 305 days of lactation (Hernandez et al. 2002, 2005; Kocak and Ekiz 2006; Miciński et al. 2009; Singh et al. 2011) or even total MY adjusted for the initial 120 days of lactation (Olechnowicz and Jaśkowski 2010).

It should be noted that evaluating total milk yield from an entire lactation may not be a favorable approach to detect real MY differences between HD-affected and healthy cows, as cows usually do not have 305 days of HD. Human interventions may eliminate both HD and MY losses. For example, MY means of 30.57 ± 0.036 (≥ 3 weeks before diagnosis), 27.52 ± 0.356 (within the week of diagnosis) and 30.42 ± 0.372 (≥ 4 weeks after diagnosis) were reported by Kocak and Ekiz (2006), suggesting that post-diagnostic treatment has the potential to restore MY to pre-diagnostic levels. Therefore, total MY in an entire lactation is a mix of healthy and HD-affected periods. If the HD-affected periods are short, the impact on 305 days MY will be insignificant. Therefore, measuring MY close to the HD-diagnostic day has greater potential to identify a significative effect of HD on MY.

### Lactation order

Lactation order was analyzed in all previous studies. However, some studies calculated HD effects separately for primiparous and multiparous cows (Charfeddine and Pérez-Cabal 2017; Kocak and Ekiz 2006; Miciński et al. 2009; Onyiro et al. 2008; Rajala-Schultz et al. 1999; Warnick et al. 2001). Other studies (Table 1) fitted lactation order into the model but did not estimate HD effect within lactation order. Among the studies that estimated the HD effects within lactation order, there was no consensus regarding the HD impacts. A study found no significant difference between primiparous and multiparous cows in the HD effect on MY over the lactation period (Kocak and Ekiz 2006). Three studies found significant effects mainly in multiparous (Charfeddine and Pérez-Cabal 2017; Miciński et al. 2009; Onyiro et al. 2008). Two studies found significant effects in both primiparous and multiparous cows (Rajala-Schultz et al. 1999; Warnick et al. 2001). Primiparous cows have lower physiological maturity than multiparous cows and, consequently, on average, also produce less milk than multiparous cows (Munoz-Boettcher et al. 2025). There is also evidence that cows with a higher number of lactations are more susceptible to HD. For example, Patoliya et al. (2024) reported a HD incidence rate of 6.8% in primiparous cows and 39.6% in cows with more than four lactations. Assuming that the lactation order is associated with both the MY and HD incidence rates, it is recommended that the HD effect be fitted within the lactation order when possible.

### Days in milk

Days in milk (DIM) was another fixed effect found in all previous studies, except Singh et al. (2011) and Miciński et al. (2009), as DIM is a key determinant factor affecting milk yield. The lactation curve ascends from the initial phase until reaching the lactation peak, usually between 50 and 100 days, and then descends until the end of lactation (Li et al. 2022). It must be noted that some studies have shown a higher HD incidence in the early lactation period (Green et al. 2002; Hernandez et al. 2005; Kocak and Ekiz 2006; Olechnowicz and Jaśkowski 2010; Warnick et al. 2001), which may be associated with physiological challenges that cows face during the transition period (Calderon and Cook 2011). Therefore, fitting DIM in the model allows to control the MY variations across lactation curve.

### Calving season

Many studies fitted calving season in the statistical model (Charfeddine and Pérez-Cabal 2017; Green et al. 2002; Hernandez et al. 2002, 2005; Olechnowicz and Jaśkowski 2010; Onyiro et al. 2008; Rajala-Schultz et al. 1999; Singh et al. 2011; Warnick et al. 2001), but none of them reported the HD effect on MY within calving season classes. The seasons of the year can affect MY by influencing several factors, for instance, thermal stress (Lim et al. 2021) and food availability (Timlin et al. 2021). Moreover, the season can also affect HD incidence rates. For instance, Jewell et al. (2021) observed a higher SU incidence in the spring/summer period, when cows had more access to pasture, than in the fall/winter. So, it is recommended to fit calving season in the model to adjust for any possible effect of this source of variation on MY. In addition, the calving season effect must be further studied, mainly if environmental factors are not artificially controlled throughout the seasons, for instance, in more extensive dairy systems.

### Herd effect

Some studies used data from a single herd (Bicalho et al. 2008; Hernandez et al. 2002; Kocak and Ekiz 2006; Miciński et al. 2009; Olechnowicz and Jaśkowski 2010; Onyiro et al. 2008; Pavlenko et al. 2011; Singh et al. 2011). Other studies used data from multiple herds and fitted the herd either as a random (Amory et al. 2008; Charfeddine and Pérez-Cabal 2017; King et al. 2017; Rajala-Schultz et al. 1999; van den Borne et al. 2022) or fixed effect (Green et al. 2002, 2010). Warnick et al. (2001) analyzed two herds separately and consequently did not fit the herd effect.

When data from two or more herds are simultaneously analyzed, the herd factor must be fitted into the model to account for this source of variation, which is caused by intrinsic farm characteristics. Health management, nutritional quality, and facilities may vary across herds, consequently impacting both MY and HD incidence. For instance, cows feeding rich forage diets have fewer HD incidences than cows feeding rich concentrate diets (Onyiro et al. 2008). It is also known that the proportion forage and concentrate also greatly influences MY. When the herd factor is fitted to the model, the option as a random effect seems more coherent, since the herds in each study are only a random sample of the many herds that could potentially be the target. Furthermore, the herd effect is not constant, as it can vary depending on possible farm management modifications over time.

### Body condition score

Two studies analyzed the body condition score (BCS) as a fixed effect (King et al. 2017; Onyiro et al. 2008). BCS has been reported to be non-linearly associated with MY, where increasing score values ​​up to 3.5 (on a scale of 1 to 5) are accompanied by increases in MY, but scores above 3.5 decrease MY (Roche et al. 2007). This effect was also observed by King et al. (2017), where cows with scores ≤ 2.5 and 3.0 produced, on average, 3.00 ± 0.78 and 2.44 ± 0.57 kg/day more than cows with scores ≥ 3.5, respectively. Moreover, Onyiro et al. (2008) reported a quadratic association of BCS with lameness score of primiparous and multiparous cows. Therefore, whenever possible, future studies must fit BCS as a fixed effect in the model when evaluating the HD impact on MY in dairy cows.

### Cow’s age at calving

Cow’s age at calving was analyzed as a fixed effect in two studies (Charfeddine and Pérez-Cabal 2017; Onyiro et al. 2008), as it can affect both MY and HD incidence. Adjusting this effect could be important, asPatoliya et al. (2024) reported a HD incidence of 5.7% in cows up to four years old and 25% for cows over this age. However, calving order and cow age at calving may be strongly correlated, and a bias due to a confounding effect is expected if both are fitted simultaneously into the model. This is especially true in more intensive dairy systems, where no-pregnant cows are quickly culled, which leads to a strong correlation between calving order and cow’s age.

### Hoof disorder types

Some studies estimated MY differences between HD-affected and healthy cows within specific HD lesion types (Amory et al. 2008; Charfeddine and Pérez-Cabal 2017; Green et al. 2010; Hernandez et al. 2002; Pavlenko et al. 2011; Warnick et al. 2001). Warnick et al. (2001) observed reduced MY in cows affected by hoof abscess (characterized by a pus-filled cavity in the white line or foot sole); SU (characterized by degenerative or necrotic defects in the sole near the sole-heel junction); HR (characterized by swelling of the soft tissues of the foot above the coronary band); and DD (characterized by ulcerative or proliferative lesions of the toes or interdigital region with a granular, red or gray surface). The greatest MY losses were caused by SU (​​between − 0.9 and − 2.5 kg/day), while HR resulted in smaller MY losses (between − 0.4 and − 1.2 kg/day).

Hernandez et al. (2002) also evaluated HR, DD, and a third group of cows affected by SU or WLD (characterized by the separation of the hoof wall from the sole in the region known as the white line). In this study, only HR-affected cows had significant MY losses (-855 kg/305-day). Amory et al. (2008) reported MY losses in both SU (between − 0.93 and − 1.75 kg/d) and WLD (between − 0.64 and − 2.18 kg/d) affected cows. Green et al. (2010) evaluated SU, WLD, DD and DS (characterized by two or more layers of sole developing within a single hoof, creating a thickened or separated area) affected cows. They reported significant MY losses only for SU (between − 1.27 and − 2.05 kg/day) and DS (-1.76 kg/day) affected cows. Pavlenko et al. (2011) evaluated DD and SU affected cows and reported significant MY losses only in DD-affected cows (-5.5 kg/day), while Charfeddine and Pérez-Cabal (2017) found significant MY losses in DD (-0.52 to -0.90 kg/day), SU (-0.93 to -2.48 kg/day) and WLD (-0.65 to -2.66 kg/day) affected cows. As noted, there was no consensus across studies, i.e., the HD effect size varied in magnitude and was not always significant. This lack of consensus may result from differences between studies, such as HD severity (mild, moderate, or severe), experimental design, or sampling size.

### Time between MY loss record and HD diagnosis

MY losses can be estimated at different times before and after the HD diagnosis day. Significant losses were found at 14 days (Rajala-Schultz et al. 1999), 28 days (Charfeddine and Pérez-Cabal 2017), and four months(Green et al. 2002) before HD diagnosis. These results suggest that HD, even in a sub-clinical condition, may result in MY losses. Previous studies also observed significant MY losses at 21 days (Warnick et al. 2001), 28 days (Charfeddine and Pérez-Cabal 2017), 42 days (Rajala-Schultz et al. 1999), and five months(Green et al. 2002) after diagnosis. These results suggest that even treated cows continue to experience MY losses for some time.Pavlenko et al. (2011) reported significant losses in DD-affected cows even 5 to 6 weeks post-treatment, whileGreen et al. (2010) reported significant losses up to one-month post-treatment of SU-affected cows.

### Mean differences

**Fig. 2:**
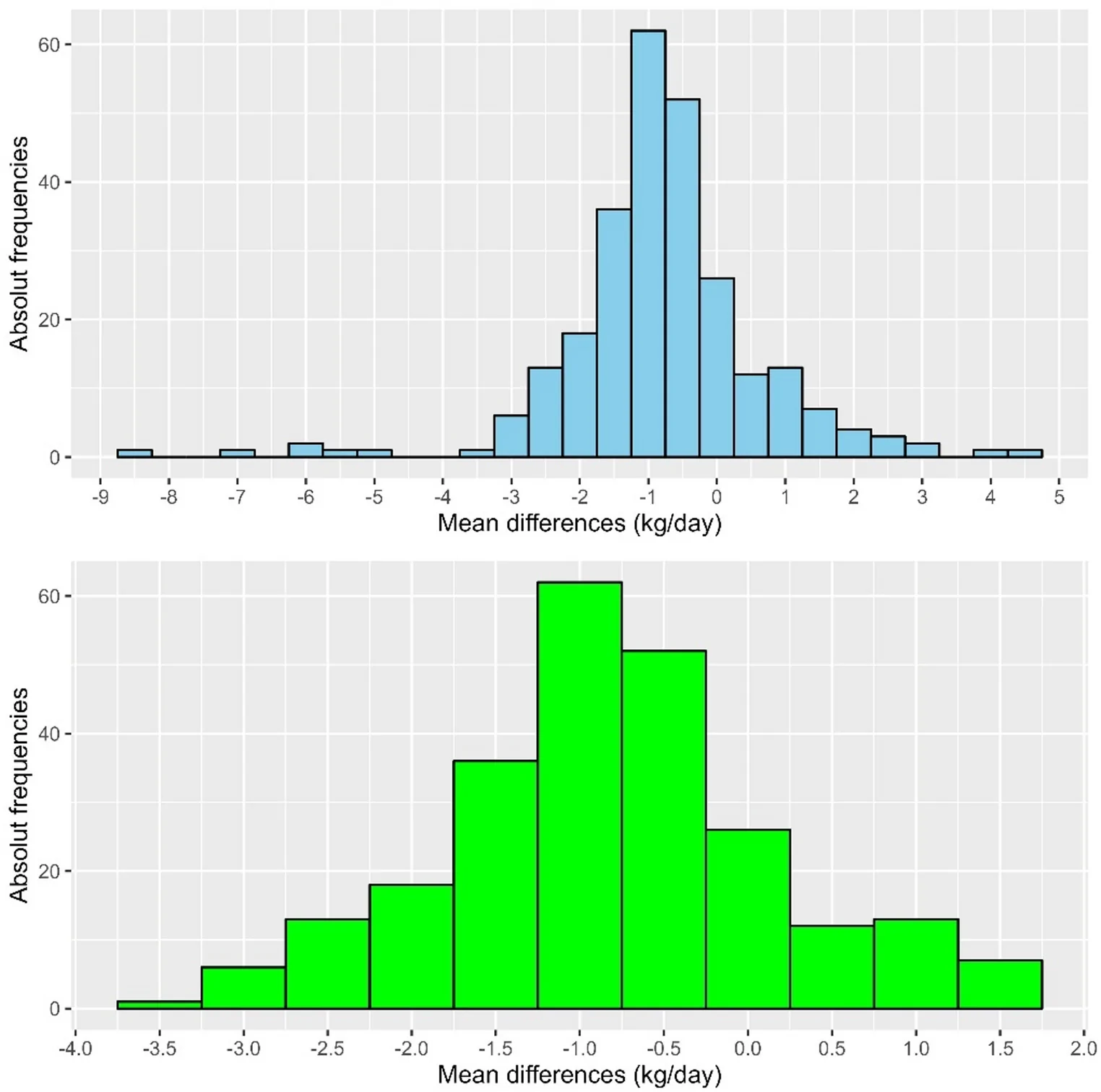
Distribution of mean differences (kg/day) between HD-affected and healthy cows with all estimates (upper plot) and after removing outliers (lower plot)

The present review identified 263 estimates of mean differences (MD) between MY from HD-affected and healthy cows (Fig. 2). The MD units were not the same across studies, so all of them were transformed into kg/day to perform descriptive statistical analyses. The MD ranged from − 8.72 kg/day to 4.56 kg/day, and both these values were reported by Hernandez et al. (2005). The mean, median, and SD estimates were − 0.79 kg/day, -0.80 kg/day, and 1.48 kg/day, respectively. Then, 17 MD estimates were found outside the lower and upper bounds of the boxplot, which were assumed to be outliers and removed. After removing outliers, MD estimates ranged from − 3.33 kg/day (Miciński et al. 2009) to 1.65 kg/day (Green et al. 2010). The mean, median, and SD estimates were − 0.81, -0.83, and 0.98 kg/day, respectively. Even removing potential outliers, the results suggest a large variation in the effect size of HD on MY across studies, including positive impacts. This variation reinforces the importance of performing meta-analyses to obtain more robust pooled estimates that better characterize the impacts of HD on MY.

### Meta-analysis

Although there are few experimental groups in previous studies, multiple estimates of HD effect on MY were reported in some studies (Amory et al. 2008; Charfeddine and Pérez-Cabal 2017; Green et al. 2002, 2010; Kocak and Ekiz 2006; Pavlenko et al. 2011; Rajala-Schultz et al. 1999; Warnick et al. 2001). This is a consequence of the study design. For instance, Rajala-Schultz et al. (1999) analyzed six times (two before and four after diagnosis day) in four lactation classes and consequently estimated 24 HD effects on MY. Meta-analysis assumes that the effect size estimates ${(\widehat{\theta}}_{k})$ are independent, i.e., they had origin in different populations. Therefore, the multiple HD effects across time within the same lactation order (Amory et al. 2008; Bicalho et al. 2008; Charfeddine and Pérez-Cabal 2017; Green et al. 2002; Green et al. 2010; Pavlenko et al. 2011; Rajala-Schulyz et al., 1999; Warnick et al. 2001) were assumed to be correlated since the same population was used to obtain the HD effects. To account for meta-analysis assumptions, we just used only one HD effect estimated closest to the diagnostic time, while HD effects in other time points were excluded.

**Fig. 3:**
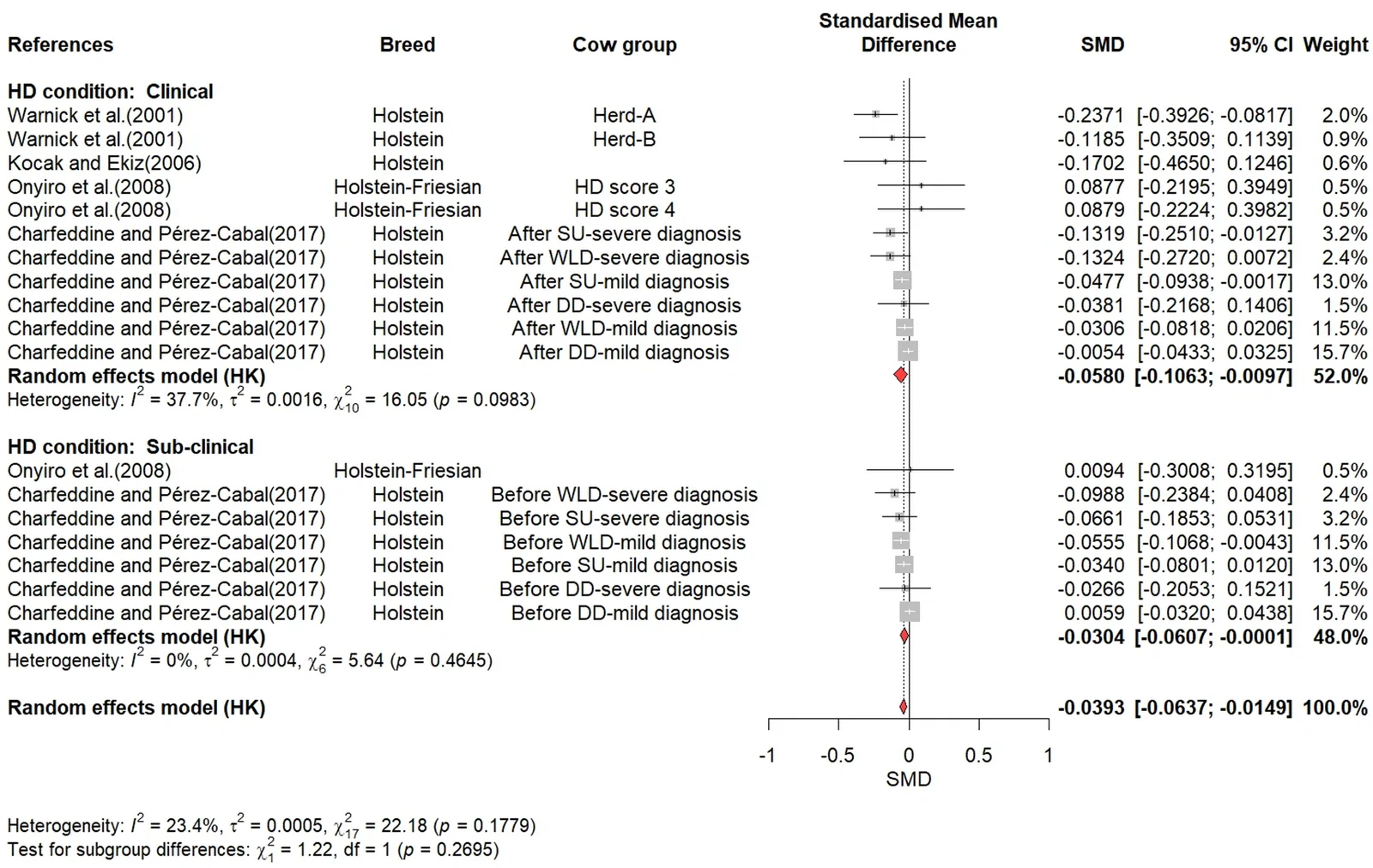
Meta-analyses of standardized mean differences (SMD) recorded in primiparous cows

For primiparous cows with sub-clinical and clinical HD conditions, respectively, seven and 11 SMD estimates were used in the meta-analysis (Fig. 3). These SMDs had normal distribution (p-value = 0.7721 in the Shapiro-Wilk test) with values ranging from − 0.2371 to 0.0879 and a mean of -0.0557 ± 0.0196. All estimates used in clinical and subclinical primiparous cows’ meta-analyses are independent because they come from different cow groups. The estimates reported byCharfeddine and Pérez-Cabal (2017) were recorded in six cow groups, i.e., a combination of three HD lesion types (DD, SU, or WLD) and two HD lesion severities (mild or severe). The two estimates reported for Warnick et al. (2001) refer to herds A and B, whereas the two estimates reported by Onyiro et al. (2008) come from two cow groups with HD lesion scores of 3 and 4, respectively. The pooled SMD estimate for the entire primiparous SMD sample was − 0.0393 (95%CI: -0.0637 to -0.0149), while the pooled SMD estimates after separate cows in sub-clinical and clinical conditions were − 0.0304 (95%CI: -0.0607 to -0.0001) and − 0.0580 (95%CI: -0.1063 to -0.0097), respectively.

These results suggest significant MY losses in primiparous cows’ clinical and subclinical conditions since the zero value is outside the 95% CI. Although the pooled SMD estimated for clinical conditions is almost twice as high as the SMD estimated for subclinical conditions, there was no significant difference (p-value = 0.2695). The interpretation of pooled SMD estimates ​​depends on the populational SD. For example, Marumo et al. (2022) evaluated 45,593 lactation records in Holstein-Friesian primiparous cows and found an average of 30.93 kg/day with a SD of 8.22 kg/day. Therefore, by multiplying the pooled SMD estimates and their 95%CI by 8.22, one would find MD estimates of -0.25 kg/day (95% CI: -0.50 to -0.0008 kg/day) and − 0.48 kg/day (95% CI: -0.87 to -0.08 kg/day) for sub-clinical and clinical HD condition in primiparous cows, respectively.

**Fig. 4:**
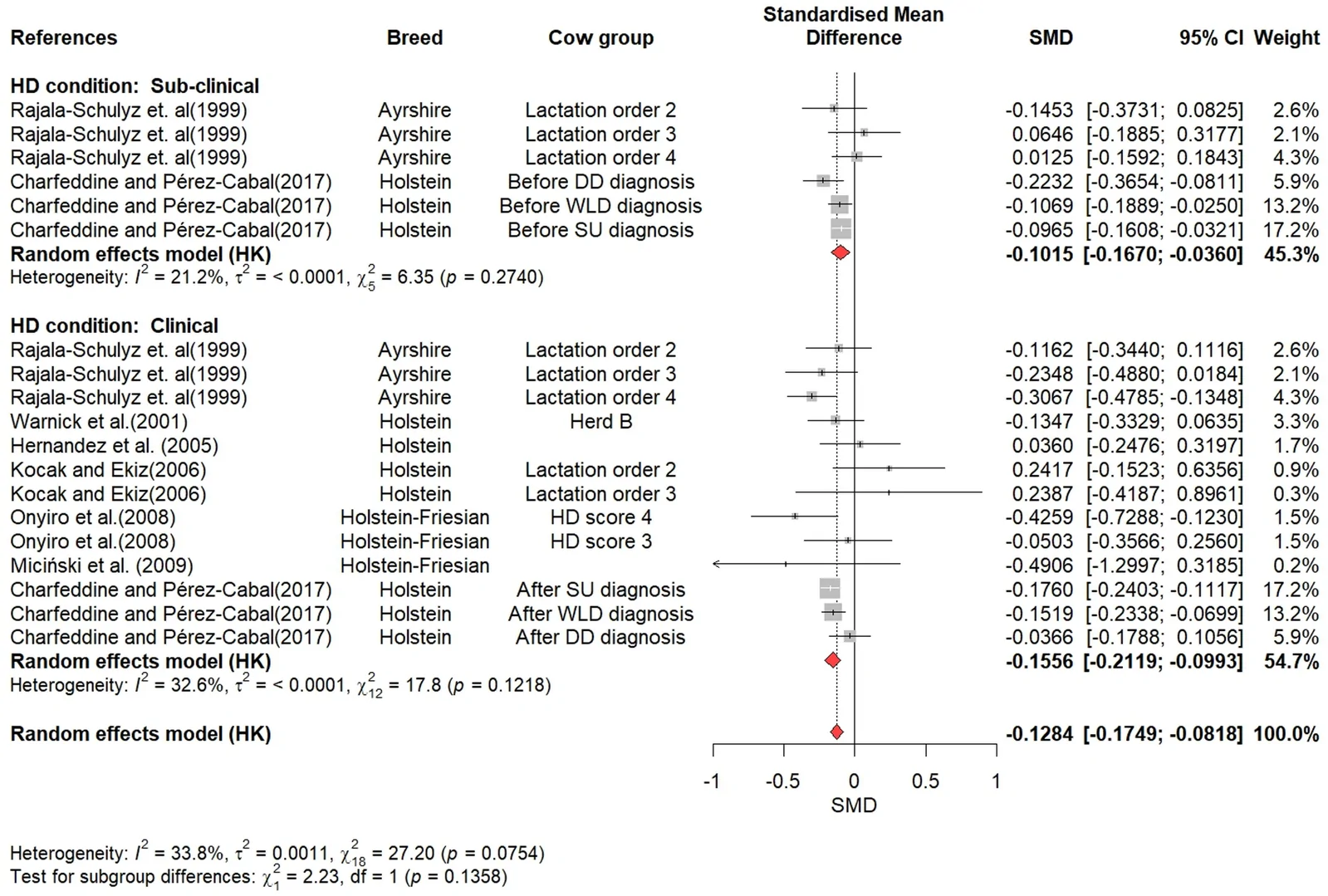
Meta-analyses of standardized mean differences (SMD) recorded in multiparous cows

For multiparous cows, six (sub-clinical) and 13 (clinical) SMD estimates were meta-analyzed (Fig. 4), which had normal distribution (p-value = 0.7266), values ranging from − 0.4906 to 0.2417, and a mean of -0.1106 ± 0.0433. All estimates used in meta-analysis of clinical and subclinical multiparous cows are independent because they come from different cow groups. Rajala-Schultz et al. 1999 reported estimates of the HD effect for three lactation orders (2nd, 3rd, and 4th). Kocak and Ekiz (2006) reported HD effects for two cow groups of lactation order 2 and 3. Onyiro et al. (2008) analyzed two cow groups with HD scores 3 and 4, respectively. While Charfeddine and Pérez-Cabal (2017) reported effects for three HD lesion types (DD, SU, and WLD) before and after HD diagnosis.

Multiparous cows with sub-clinical and clinical HD conditions had pooled SMD estimates of -0.1015 (95% CI: -0.1670 to -0.0360) and − 0.1556 (95% CI: -0.2119 to -0.0993), respectively. Therefore, there are significant milk yield losses in multiparous cows with either sub-clinical or clinical HD, as zero is outside 95% CI. These pooled SMD estimates did not differ (p-value = 0.1358), i.e., our meta-analyses do not allow us to state that the MY losses are greater or smaller in clinical or sub-clinical HD multiparous cows. Marumo et al. (2022) evaluated 79,867 lactation records of multiparous Holstein-Friesian and found an average of 38.85 kg/day with an SD of 11.01 kg/day. Therefore, for Marumo’s population, the mean MY losses in multiparous sub-clinical and clinical HD cows would be expected to be 1.12 kg/day (95% CI: 0.40 to 1.84 kg/day) and 1.71 kg/day (95% CI: 1.09 to 2.33 kg/day), respectively.

The pooled SMD estimated for primiparous and multiparous cows within sub-clinical or clinical conditions did not differ since their 95% CI overlapped. On the other hand, a significant difference between clinical HD multiparous cows (pooled SMD = -0.1556; 95% CI: -0.2119 to -0.0993) and sub-clinical HD primiparous cows (pooled SMD = -0.0304; 95%CI: -0.0607 to -0.0001) was found, as their 95% CI did not overlap. These results suggest that HD impacts on MY in multiparous and primiparous cows are similar, and differences are found only if the HD severity is different.

**Fig. 5:**
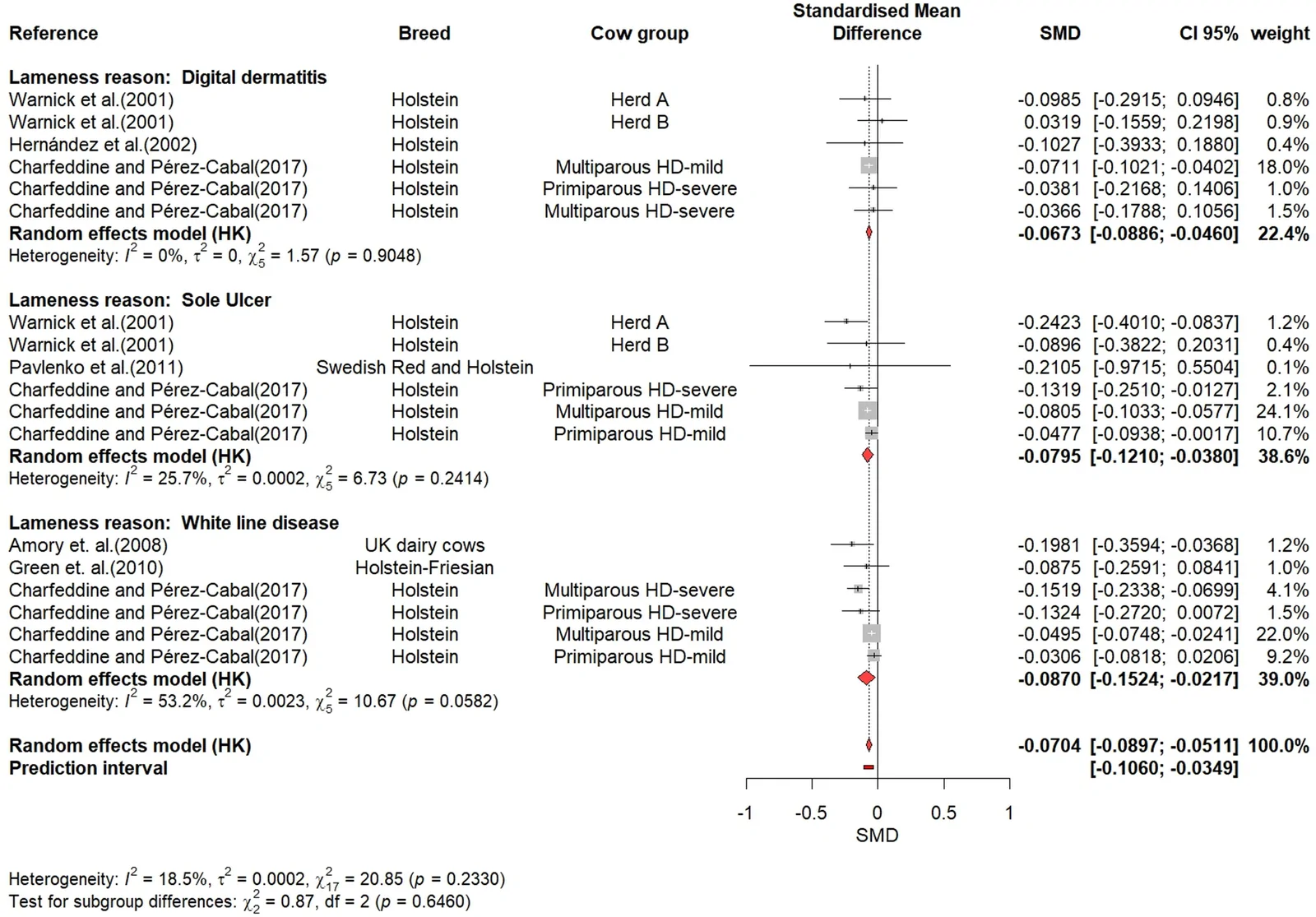
Meta-analyses of standardized mean differences (SMD) based on hoof disorder types

A subgroup meta-analysis of SMD estimates according to three HD types (DD, SU, and WLD) was also carried out (Fig. 5). The SMD estimates had a normal distribution (*p* = 0.6395), with values ​​between − 0.2423 and 0.0319 and a mean of -0.0982 ± 0.0165. All the estimates used in DD, SU and WLD meta-analyses are independent. Warnick et al. (2001) reported HD effects for herds A and B, while the estimates reported by Charfeddine and Pérez-Cabal (2017) come from cows with mild or severe HD combined with lactation orders (primiparous or multiparous). The pooled SMD were − 0.0673 (95% CI: -0.0886 to -0.0460), -0.0795 (95% CI: -0.1210 to -0.0380), and − 0.0870 (95% CI: -0.1524 to -0.0217) for DD-, SU- and WLD-affected cows, respectively. Therefore, these three HD types can cause MY losses, as the zero value is outside the 95% CI, but one can not assume that there is a difference between these pooled estimates as the Q test for subgroup differences was not significant (p-value = 0.6460).

The pooled SMD estimates in the meta-analysis of HD types were higher than in primiparous and lower than in multiparous meta-analyses, as previous studies used a mix of primiparous and multiparous cows to estimate HD effect based on HD types. A previous study analyzed daily milk yield records from 192,551 Holstein cows (lactations 1, 2, or 3), resulting in a mean of 40.8 kg/day with an SD of 10.2 kg/day (Guinan et al. 2024). Therefore, for Guinan et al. (2024) dairy cattle population, one could expect MY losses (in kg/day) in cows affected by DD, SU, and WLD of 0.69 (95% CI: 0.47 to 0.90), 0.81 (95% CI: 0.39 to 1.23), and 0.89 (95% CI: 0.22 to 1.55), respectively.

### Between study heterogeneity and publication bias

The BSH was low or moderate depending on the meta-analysis but not significant (*P* > 0.05) based on the Q-test. In primiparous cows, the I^2^ and the Q-test p-values were as follows: (a) sub-clinical (I^2^ = 0%; p-value = 0.4645), (b) clinical (I^2^ = 37.7%; p-value = 0.0983), and (c) all primiparous cows together (I^2^ = 23.4%; p-value = 0.1779); while in multiparous cows the BSH parameters were: (a) sub-clinical (I^2^ = 21.2%; p-value = 0.2740), (b) clinical (I^2^ = 32.6%; p-value = 0.1218), and (c) all multiparous cows together (I^2^ = 33.8%; p-value = 0.0754). In meta-analyses based on HD types the I^2^ and p-values were DD (I^2^ = 0%, p-value = 0.9048), SU (I^2^ = 25.7%; p-value 0.2414), WLD (I^2^ = 53.2%; p-value = 0.0582), and the joint analysis of three HD causes (I^2^ = 18.5%; p-value = 0.2330).

A moderate BSH can be assumed for WLD based on the I^2^ value, but the Q-test was insignificant even in this case. Moderate or high BSH can influence the meta-analysis results. Thus, even when BSH is not significant, adjusting the study factor as a random effect is recommended, which creates a variance component due to BSH. This variance component contributes to calculating the weights (last column on the right side of Figs. 3, 4 and 5) of each study in the meta-analysis. In addition, the Hartung-Knapp method was also used to estimate the 95% CI of the pooled estimates, which is appropriate when there is BSH (Harrer et al. 2022). Therefore, there is no reason to assume that the pooled SMD estimates are not robust based on the BSH found.

**Fig. 6:**
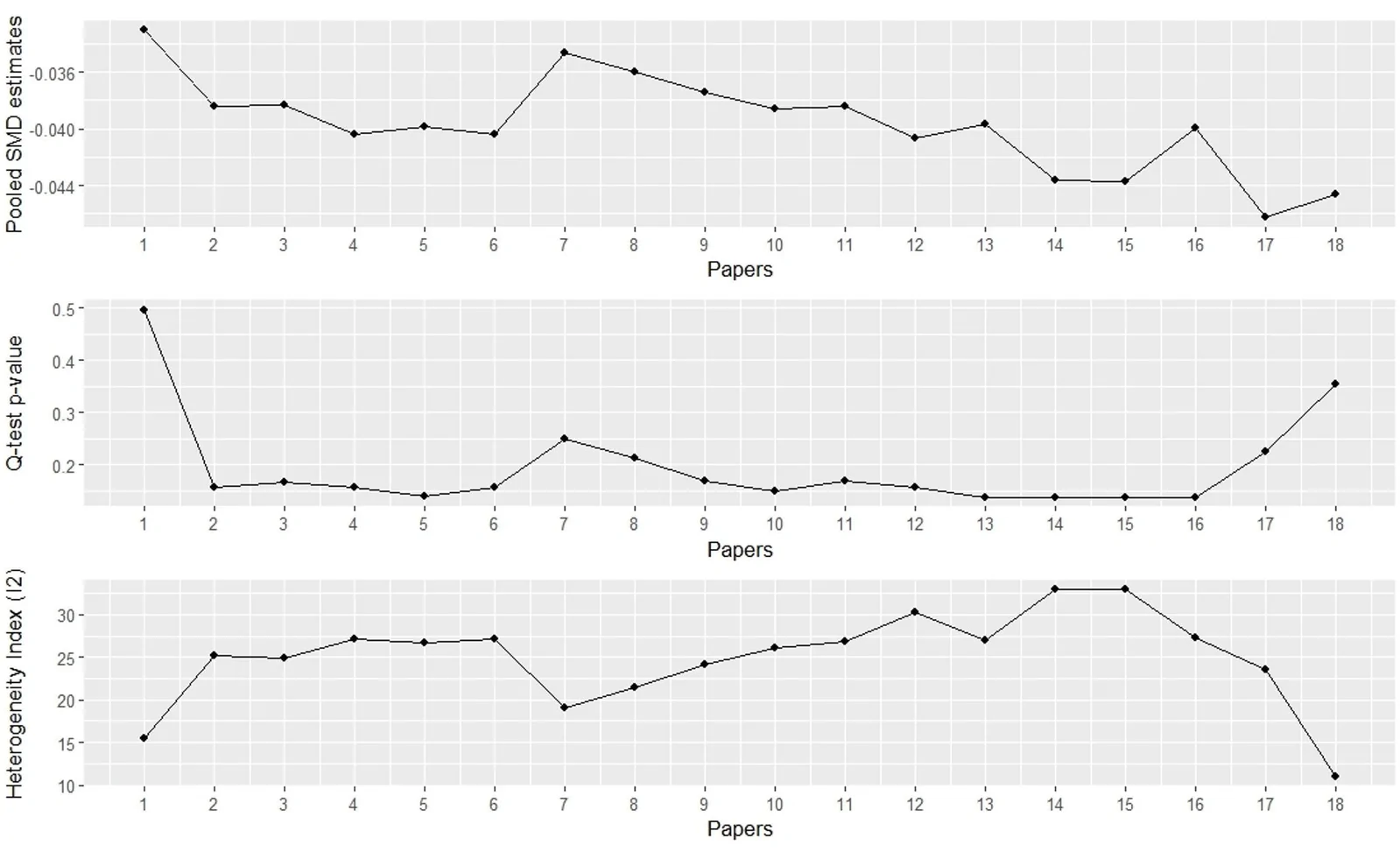
Leave-one-out results for meta-analysis based on primiparous data

**Fig. 7:**
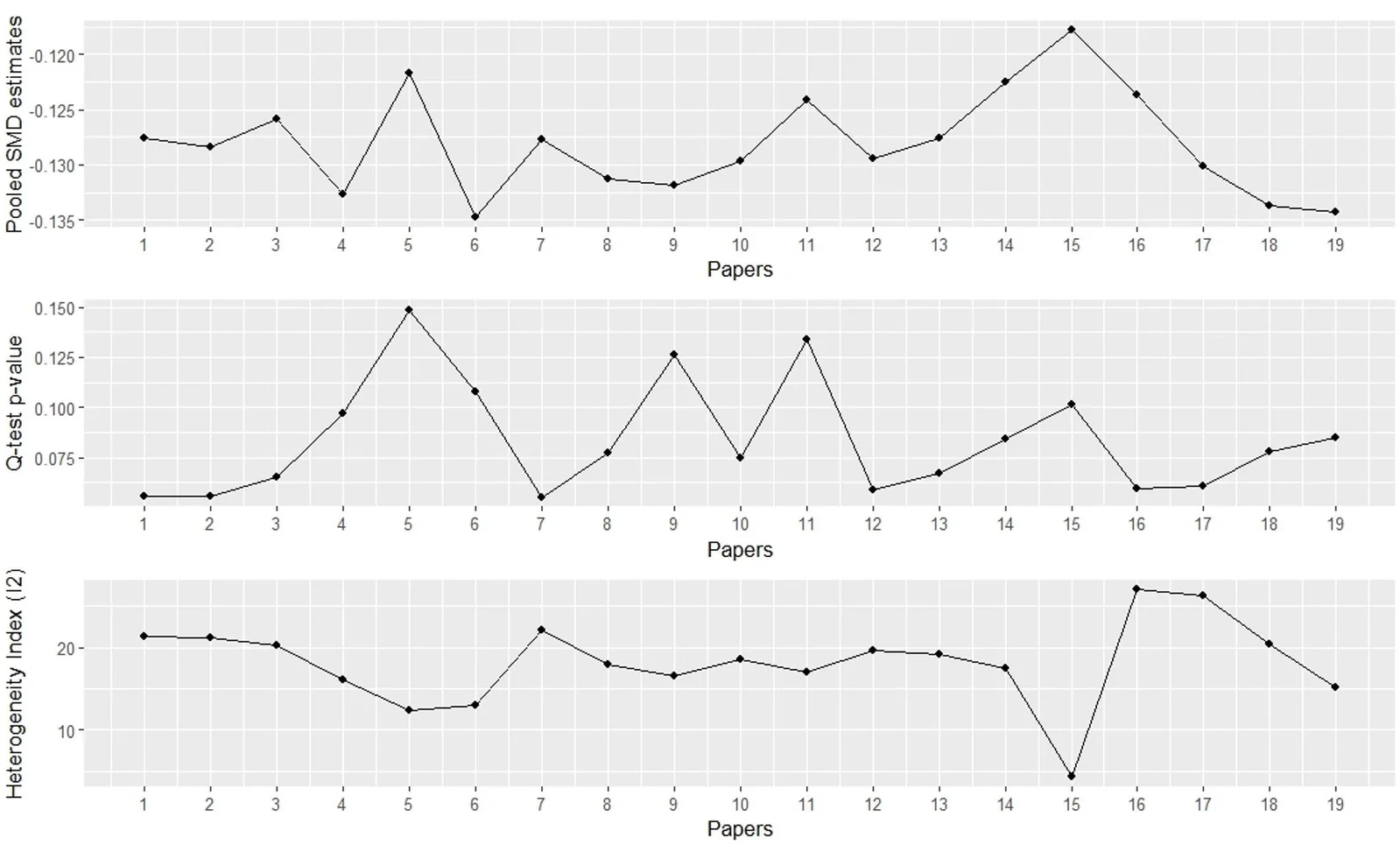
Leave-one-out results for meta-analysis based on multiparous data

**Fig. 8:**
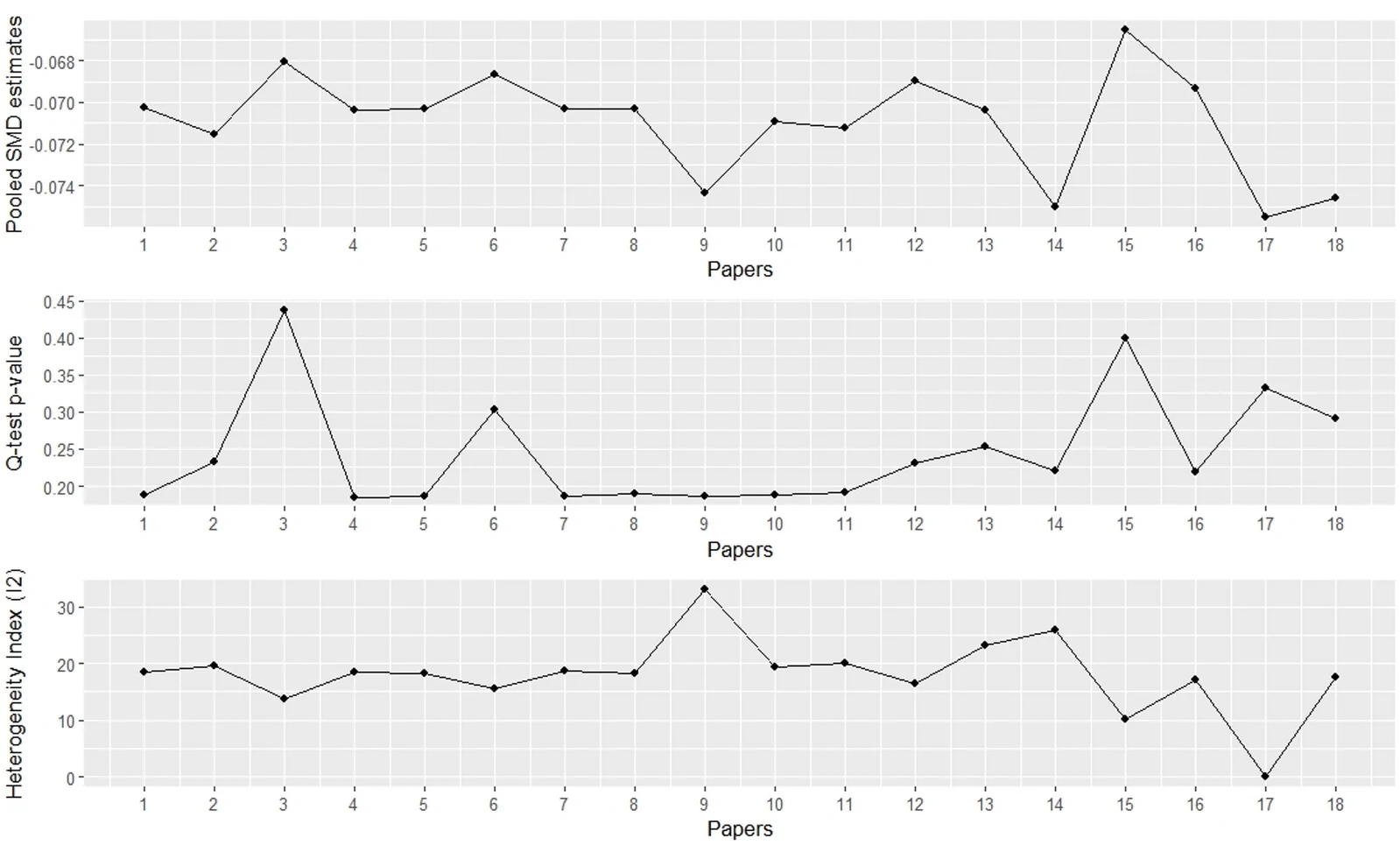
Leave-one-out results the meta-analysis based on HD types

Results of the “leave-one-out” strategy (Figs. 6, 7, and 8) suggest that removing any SMD estimate would not significantly change the pooled SMD estimates (top graph in each figure), or the Q-test p-value (middle graph in each figure), or the I^2^ (bottom graph in each figure). Furthermore, there are no outliers in the meta-analyses performed, which can be checked by looking at the (at least partially) overlap of the 95% CI of the SMD estimates with the 95% CI of the pooled SMD estimates (Figs. 3, 4 and 5).

**Fig. 9:**
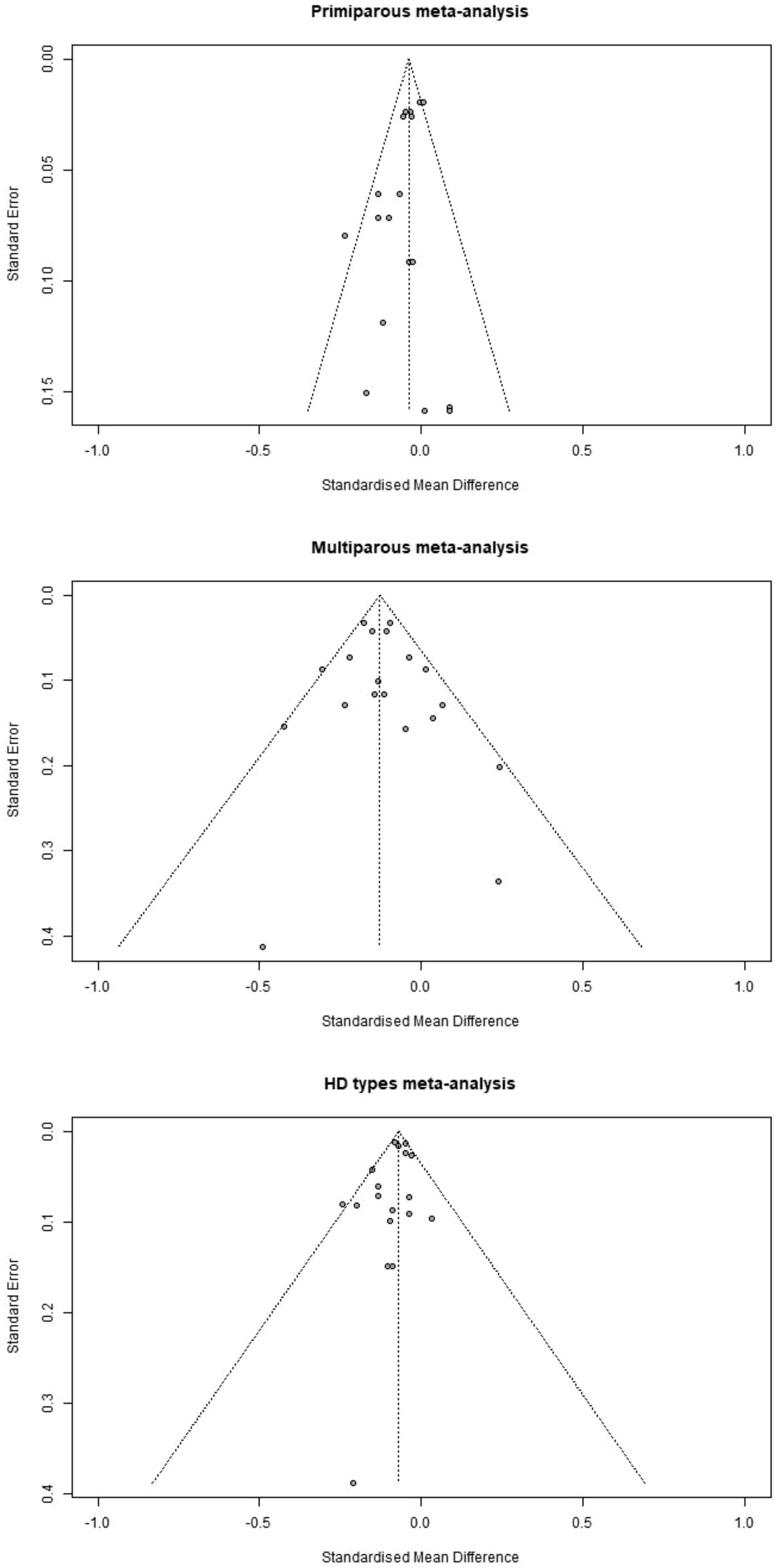
Funnel plot of the standardized mean differences (SMD) based on the three meta-analyses performed

Pooled SMD estimated by meta-analysis is a consequence of the SMD estimates reported in previous studies. Therefore, if an SMD estimate has not been published for any reason, there is what is known as publication bias. In the current study, publication bias was assessed based on a graphical analysis of funnel plots (Fig. 9), which revealed no strong asymmetry, suggesting no evidence of publication bias. Previous studies published a wide range of HD effects on MY, including positive effects, which support ​​no publication bias.

### Final considerations

The present systematic review found many estimates of the HD effect on MY in dairy cows and estimated pooled SMDs. Significant MY loss is expected in primiparous and multiparous cows in clinical or subclinical conditions. Based on the current literature, it is not possible to state that primiparous and multiparous cows with the same HD condition (sub-clinical or clinical) differ regarding milk yield losses, and the differences found in previous studies are probably a consequence of higher productivity of multiparous cows compared to primiparous. SU, WLD, and DD cause MY losses, but it can not be stated that any of these diseases have a greater negative impact than the others.

The SMD reported here are now more robust quantitative references of the negative HD impact on dairy cows’ productivity. It is important to note that the pooled SMDs depend on the intrinsic characteristics of the previous studies, which mainly evaluated Holstein-Friesian cows raised in intensive dairy systems on temperate climates. Therefore, one can not assume that these pooled SMDs are adequate for other environmental conditions (for instance, pasture-based systems) or genetic groups (for instance, zebu cattle). Future studies must be carried out to estimate the HD effects on MY in other climates, breeds, and dairy systems, allowing future meta-analysis studies to estimate pooled SMDs applied to each situation.

As in many meta-analyses in the health sciences, the current one also relies on disease diagnosis, which can sometimes be subject to interpretation. Different interpretations can generate high BSH. Here, we adopted elementary meta-analysis approaches such as: (a) fitting the study as a random effect; (b) checking for outliers and influential observations; (c) using an appropriate method to estimate the 95%CI of the pooled estimates; and (d) checking for publication bias. Therefore, the pooled estimates can be assumed as robust based on the HD effects on MY previously published. Despite this, we recommend that future studies aiming to identify HD effects on MY focus on existing literature, prioritizing more established diagnostic methods to standardize this type of study. The current systematic review might be a valuable source of information for this purpose.

## Conclusions

Significant MY loss is expected in primiparous and multiparous cows in clinical or subclinical conditions. Based on the current literature, it is not possible to state that primiparous and multiparous cows with the same HD condition (sub-clinical or clinical) differ regarding milk yield losses, and the differences found in previous studies are probably a consequence of higher productivity of multiparous cows compared to primiparous. SU, WLD, and DD cause MY losses, but it cannot be stated that any of these diseases have a greater negative impact than the others.

## Data Availability

The datasets analyzed in the current study are available in published papers cited across the manuscript and can also be found in the forest plots.

## References

[CR1] Amory JR, Barker ZE, Wright JL, Mason SA, Blowey RW, Green LE (2008) Associations between sole ulcer, white line disease and digital dermatitis and the milk yield of 1824 dairy cows on 30 dairy cow farms in England and Wales from February 2003-November 2004. Prev Vet Med 83:381–391. 10.1016/j.prevetmed.2007.09.007 18031851

[CR2] Balduzzi S, Rücker G, Schwarzer G (2019) How to perform a meta-analysis with R: A practical tutorial. Evid Based Ment Health 22:153–160. 10.1136/ebmental-2019-300117 31563865 PMC10231495

[CR3] Bicalho RC, Warnick LD, Guard CL (2008) Strategies to analyze milk losses caused by diseases with potential incidence throughout the lactation: A lameness example. J Dairy Sci 91:2653–2661. 10.3168/jds.2007-0744 18565924

[CR4] Bruijnis MRN, Beerda B, Hogeveen H, Stassen EN (2012a) Foot disorders in dairy cattle: Impact on cow and dairy farmer. Anim Welf 21:33–40. 10.7120/096272812X13345905673601 22558967

[CR5] Bruijnis MRN, Beerda B, Hogeveen H, Stassen EN (2012b) Assessing the welfare impact of foot disorders in dairy cattle by a modeling approach. Animal 6:962–970. 10.1017/S1751731111002606 22558967

[CR6] Calderon DF, Cook NB (2011) The effect of lameness on the resting behavior and metabolic status of dairy cattle during the transition period in a freestall-housed dairy herd. J Dairy Sci 94:2883–2894. 10.3168/jds.2010-3855 21605758

[CR7] Charfeddine N, Pérez-Cabal MA (2017) Effect of claw disorders on milk production, fertility, and longevity, and their economic impact in Spanish Holstein cows. J Dairy Sci 100:653–665. 10.3168/jds.2016-11434 27865503

[CR8] Cochran WG (1954) The Combination of Estimates from Different Experiments. Biometrics 10:101–129. 10.2307/3001666

[CR9] Green LE, Borkert J, Monti G, Tadich N (2010) Associations between lesion-specific lameness and the milk yield of 1,635 dairy cows from seven herds in the Xth region of Chile and implications for management of lame dairy cows worldwide. Anim Welf 19:419–427. 10.1017/s0962728600001901

[CR10] Green LE, Hedges VJ, Schukken YH, Blowey RW, Packington AJ (2002) The impact of clinical lameness on the milk yield of dairy cows. J Dairy Sci 85:2250–2256. 10.3168/jds.S0022-0302(02)74304-X 12362457

[CR11] Guinan FL, Fourdraine RH, Peñagaricano F, Weigel KA (2024) Genetic analysis of lactation consistency in US Holsteins using temporal variation in daily milk weights. J Dairy Sci 107:2194–2206. 10.3168/jds.2023-24093 37923210

[CR12] Harrer M, Cuijpers P, Furukawa TA, Ebert DD (2022) Doing Meta-Analysis in R: A Hands-On Guide. Chapman & Hall/CRC, London

[CR13] Hartung J, Knapp G (2001) A refined method for the meta-analysis of controlled clinical trials with binary outcome. Stat Med 20:3875–3889. 10.1002/sim.1009 11782040

[CR15] Hernandez JA, Garbarino EJ, Shearer JK, Risco CA, Thatcher WW (2005) Comparison of milk yield in dairy cows with different degrees of lameness. J Am Vet Med Assoc 227:1292–1296. 10.2460/javma.2005.227.1292 16266019

[CR14] Hernandez J, Shearer JK, Webb DW (2002) Effect of lameness on milk yield in dairy cows. J Am Vet Med Assoc 220:640–644. 10.2460/javma.2002.220.640 12418524

[CR16] Higgins JPT, Thompson SG (2002) Quantifying heterogeneity in a meta-analysis. Stat Med 21:1539–1558. 10.1002/sim.1186 12111919

[CR17] Jewell MT, Cameron M, McKenna SL, Cockram MS, Sanchez J, Keefe GP (2021) Relationships between type of hoof lesion and behavioral signs of lameness in Holstein cows housed in Canadian tiestall facilities. J Dairy Sci 104(1):937–946. 10.3168/jds.2019-17296 33189286

[CR18] King MTM, LeBlanc SJ, Pajor EA, DeVries TJ (2017) Cow-level associations of lameness, behavior, and milk yield of cows milked in automated systems. J Dairy Sci 100:4818–4828. 10.3168/jds.2016-12281 28434734

[CR19] Kocak O, Ekiz B (2006) The effect of lameness on milk yield in dairy cows. Acta Vet Brno 75. 10.2754/avb200675010079

[CR21] Lim DH, Mayakrishnan V, Ki KS, Kim Y, Kim T-I (2021) The effect of seasonal thermal stress on milk production and milk compositions of Korean Holstein and Jersey cows. Anim Biosci 34:567–574. 10.5713/ajas.19.0926 32777906 PMC7961267

[CR20] Li M, Rosa GJM, Reed KF, Cabrera VE (2022) Investigating the effect of temporal, geographic, and management factors on US Holstein lactation curve parameters. J Dairy Sci 105:7525–7538. 10.3168/jds.2022-21882 35931477

[CR22] Marumo JL, Lusseau D, Speakman JR, Mackie M, Hambly C (2022) Influence of environmental factors and parity on milk yield dynamics in barn-housed dairy cattle. J Dairy Sci 105:1225–1241. 10.3168/jds.2021-20698 34802739

[CR23] Miciński J, Pogorzelska J, Barański W, Kalicka B (2009) Effect of disease incidence on the milk performance of high-yielding cows in successive lactations. Pol J Nat Sci 24:102–112. 10.2478/v10020-009-0010-1

[CR24] Munoz-Boettcher P, Velez J, Rodriguez N, Klaas IC, Pinedo P (2025) Milking behavior and performance of primiparous and multiparous Holstein, Jersey, and Holstein × Jersey crossbred cows in a batch milking system with automatic milking units. J Dairy Sci 108:4248–4262. 10.3168/jds.2024-26078 40043762

[CR25] Olechnowicz J, Jaśkowski JM (2010) Impact of clinical lameness, calving season, parity, and month of lactation n milk, fat, protein, and lactose yields during early lactation of dairy cows. Bull Vet Inst Pulawy 54:605–610. 10.13140/2.1.2419.2964

[CR26] Onyiro OM, Offer J, Brotherstone S (2008) Risk factors and milk yield losses associated with lameness in Holstein-Friesian dairy cattle. Animal 2:1230–1237. 10.1017/S1751731108002279 22443736

[CR27] Ouzzani M, Hammady H, Fedorowicz Z, Elmagarmid A (2016) Rayyan-a web and mobile app for systematic reviews. Syst Rev 5:210. 10.1186/s13643-016-0384-4 27919275 PMC5139140

[CR38] Page MJ, McKenzie JE, Bossuyt PM, Boutron I, Hoffmann TC, Mulrow CD, Shamseer L, Tetzlaff JM, Akl EA, Brennan SE, Chou R, Glanville J, Grimshaw JM, Hróbjartsson A, Lalu MM, Li T, Loder EW, Mayo-Wilson E, McDonald S, McGuinness LA, Stewart LA, Thomas J, Tricco AC, Welch VA, Whiting P, Moher D (2021) The PRISMA 2020 statement: an updated guideline for reporting systematic reviews. BMJ 372:n71. 10.1136/bmj.n71 PMC8005924 33782057

[CR28] Patoliya P, Kataktalware MA, Raval K, Devi LG, Sivaram M, Praveen S, Meena P, Jeyakumar S, Mech A, Ramesha KP (2024) Assessing lameness prevalence and associated risk factors in crossbred dairy cows across diverse management environments. BMC Vet Res 20:229. 10.1186/s12917-024-04093-w 38796437 PMC11127402

[CR29] Pavlenko A, Bergsten C, Ekesbo I, Kaart T, Aland A, Lidfors L (2011) Influence of digital dermatitis and sole ulcer on dairy cow behaviour and milk production. Animal 5:1259–1269. 10.1017/S1751731111000255 22440178

[CR30] Rajala-Schultz PJ, Gröhn YT, McCulloch CE (1999) Effects of milk fever, ketosis, and lameness on milk yield in dairy cows. J Dairy Sci 82:288–294. 10.3168/jds.S0022-0302(99)75235-5 10068950

[CR31] Roche JR, Lee JM, Macdonald KA, Berry DP (2007) Relationships among body condition score, body weight, and milk production variables in pasture-based dairy cows. J Dairy Sci 90:3802–3815. 10.3168/jds.2006-740 17638991

[CR32] Singh Y, Lathwal SS, Chakravarty AK, Gupta AK, Mohanty TK, Raja TV, Dangi RL, Roy BK (2011) Effect of lameness (hoof disorders) on productivity of Karan Fries crossbred cows. Anim Sci J 82:169–174. 10.1111/j.1740-0929.2010.00800.x 21269376

[CR33] Thomsen PT, Munksgaard L, Togersen FA (2008) Evaluation of a lameness scoring system for dairy cows. J Dairy Sci 91:119–126. 10.3168/jds.2007-0496 18096932

[CR34] Timlin M, Tobin JT, Brodkorb A, Murphy EG, Dillon P, Hennessy D, O’donovan M, Pierce KM, O’callaghan TF (2021) The impact of seasonality in pasture-based production systems on milk composition and functionality. Foods 10:607. 10.3390/foods10030607 33809356 PMC7998991

[CR35] van den Borne BHP, Di Giacinto Villalobos AM, Hogeveen H (2022) Disentangling the relationships between lameness, milking frequency and milk production in Dutch dairy herds using an automatic milking system. Prev Vet Med 208:105733. 10.1016/j.prevetmed.2022.105733 35961128

[CR36] Viechtbauer W (2010) Conducting Meta-Analyses in R with the metafor Package. J Stat Softw 36:1–46. 10.18637/jss.v036.i03

[CR37] Warnick LD, Janssen D, Guard CL, Gröhn YT (2001) The effect of lameness on milk production in dairy cows. J Dairy Sci 84:1988–1997. 10.3168/jds.S0022-0302(01)74642-5 11573778

